# The Analysis of the BpBZR1 Gene Family Indicates That BpBEH4-2 Positively Regulates the Cold Tolerance of *Bougainvillea*

**DOI:** 10.3390/plants15213278

**Published:** 2026-10-23

**Authors:** Ping Zhou, Xiumei Hou, Jingwen Li, Mengyu Ren, Xinyao Ye, Xue Huang, Lihui Zeng

**Affiliations:** 1 Institute of Genetics and Breeding in Horticultural Plants, College of Horticulture, Fujian Agriculture and Forestry University, Fuzhou 350002, China; 18582197443@163.com (X.H.); rmy3406212002@outlook.com (M.R.); hammer202312@163.com (X.Y.); 17875660331@163.com (X.H.); 2 Key Laboratory of Ministry of Education for Genetics, Breeding and Multiple Utilization of Crops, Fujian Agriculture and Forestry University, Fuzhou 350002, China; lijw@fafu.edu.cn

**Keywords:** cold stress, *Bougainvillea*, brassinosteroid, BpBEH4-2

## Abstract

Cold stress represents one of the major limiting factors restricting the large-scale cultivation and industrial application of *Bougainvillea* in northern cold regions. Brassinosteroids (BRs) are essential steroid phytohormones that regulate plant tolerance to diverse abiotic stresses; however, their regulatory roles and molecular mechanisms underlying cold response in *Bougainvillea* remain unclear. In this study, exogenous 24-epibrassinolide (EBR) application significantly enhanced the cold tolerance of *Bougainvillea*, while the BR biosynthesis inhibitor brassinazole (Brz) significantly reduced cold resistance. EBR treatment can reduce malondialdehyde (MDA) accumulation, increase soluble sugar content, and enhance antioxidant enzyme activity. Furthermore, we systematically identified seven members of the BpBZR1 transcription factor family. qRT-PCR results showed that *BpBZR1s* are responsive to EBR treatment. Notably, BpBEH4-2 can be significantly induced under cold stress. Heterologous overexpression of *BpBEH4-2* in *Arabidopsis thaliana* improved plant chilling tolerance and activated the expression of cold-responsive genes. These results indicated that the BR signaling pathway positively regulates cold tolerance in *Bougainvillea*, and BpBEH4-2 serves as a critical regulatory gene in cold stress adaptation. This study provides new insights into BR-mediated cold resistance mechanisms and supplies promising gene resources for cold-tolerant *Bougainvillea* breeding.

## 1. Introduction

*Bougainvillea*, native to South America, is a highly regarded ornamental plant species worldwide. Certain varieties of *Bougainvillea* produce phytochemicals with antimicrobial activity, making them valuable not only for ornamental purposes but also for preventive applications [1,2]. Furthermore, *Bougainvillea* exhibits drought tolerance, and its ecotypes include spiny trees, shrubs, and vines, enabling the plant to adapt to diverse geographical environments [3]. *Bougainvillea* is widely cultivated across various countries and regions, such as Ethiopia, Indonesia, Greece, and the Mediterranean coast. In China, its cultivation is primarily concentrated in southern regions, including Macao, Hong Kong, Yunnan, Hainan, Taiwan, Guangdong, and Fujian [4]. Due to its vibrantly colored bracts, long flowering period, drought tolerance, and ease of cultivation, *Bougainvillea* has attracted increasing interest, leading to its introduction for landscaping use even in cities of northern China. However, *Bougainvillea* thrives in sunny environments and is intolerant of cold stress. Low temperatures significantly impact its normal growth; growth generally ceases at 10 °C, accompanied by a loss of leaf sheen. Bracts begin to abscise at temperatures below 5 °C, while exposure to sub-zero temperatures causes frost damage, which can be severe enough to result in plant death [5,6,7]. These characteristics severely restrict its cultivation in cold regions. Consequently, studying the cold resistance of *Bougainvillea* is of great significance for promoting its open-field cultivation in cold environments.

Brassinosteroid (BR) play a crucial role in plant responses to cold stress [8]. For example, exogenous application of BRs has been shown to promote growth recovery after cold exposure in maize [9]. BR treatment can also reduce cold injury in *Arabidopsis* [10], banana [11] and loquat [12]. Two BR signaling-deficient mutants (*bri1-1* and *bri1-301*) exhibited significantly reduced cold tolerance, with markedly lower survival rates under low temperatures [13].

The BRASSINAZOLE-RESISTANT (BZR1) transcription factor family plays a central role in BR signal transduction [14]. Studies have demonstrated that the BZR1 transcription factor plays a positive regulatory role in plant responses to low-temperature stress. For example, in *Arabidopsis*, BR enhance freezing tolerance by facilitating BZR1-mediated regulation of *CBF* expression [15]. Under cold conditions, BZR1 upregulates the expression of its target gene *BAM* (β-amylase), which is involved in starch hydrolysis, leading to the accelerated degradation of sucrose and starch [16]. This finding provides new insights into improving freezing tolerance in foxtail millet through the crosstalk between BR signaling and starch/sucrose metabolism under cold stress. Additionally, the BZR1 transcription factor often acts as a key hub integrating BR and other hormonal signals to regulate plant adaptation to low temperatures. Overexpression of *BZR1* in tomato enhances ABA levels by inducing the expression of *NCED1*, a gene involved in ABA biosynthesis. The study indicates that the cold resistance mediated by BR is highly dependent on the level of ABA. Both low-temperature stress and BR treatment significantly suppress the negative regulator BIN2 in the BR signaling pathway, thereby releasing BZR1 to perform its functional roles [17].

In this study, EBR and Brz were performed to investigate the regulatory effect of BR on cold tolerance in *Bougainvillea*. Meanwhile, members of BZR1 transcription factor family were identified from the *Bougainvillea* genome, and the expression profiles of these family genes under cold stress were characterized. The results revealed that *BpBEH4-2* can be induced by cold stress. Furthermore, functional validation of BpBEH4-2 was carried out using a heterologous overexpression system in *Arabidopsis thaliana*. Collectively, this study demonstrates that BRs positively regulate cold stress responses in *Bougainvillea*. The cold-responsive properties of the BpBZR1 gene family were systematically characterized. The results provide a theoretical basis for further elucidating the molecular regulatory mechanisms by which the BpBZR1 family mediates cold stress responses in *Bougainvillea*.

## 2. Results

### 2.1. Exogenous EBR Application Improves Cold Tolerance in Bougainvillea

**Figure 1:**
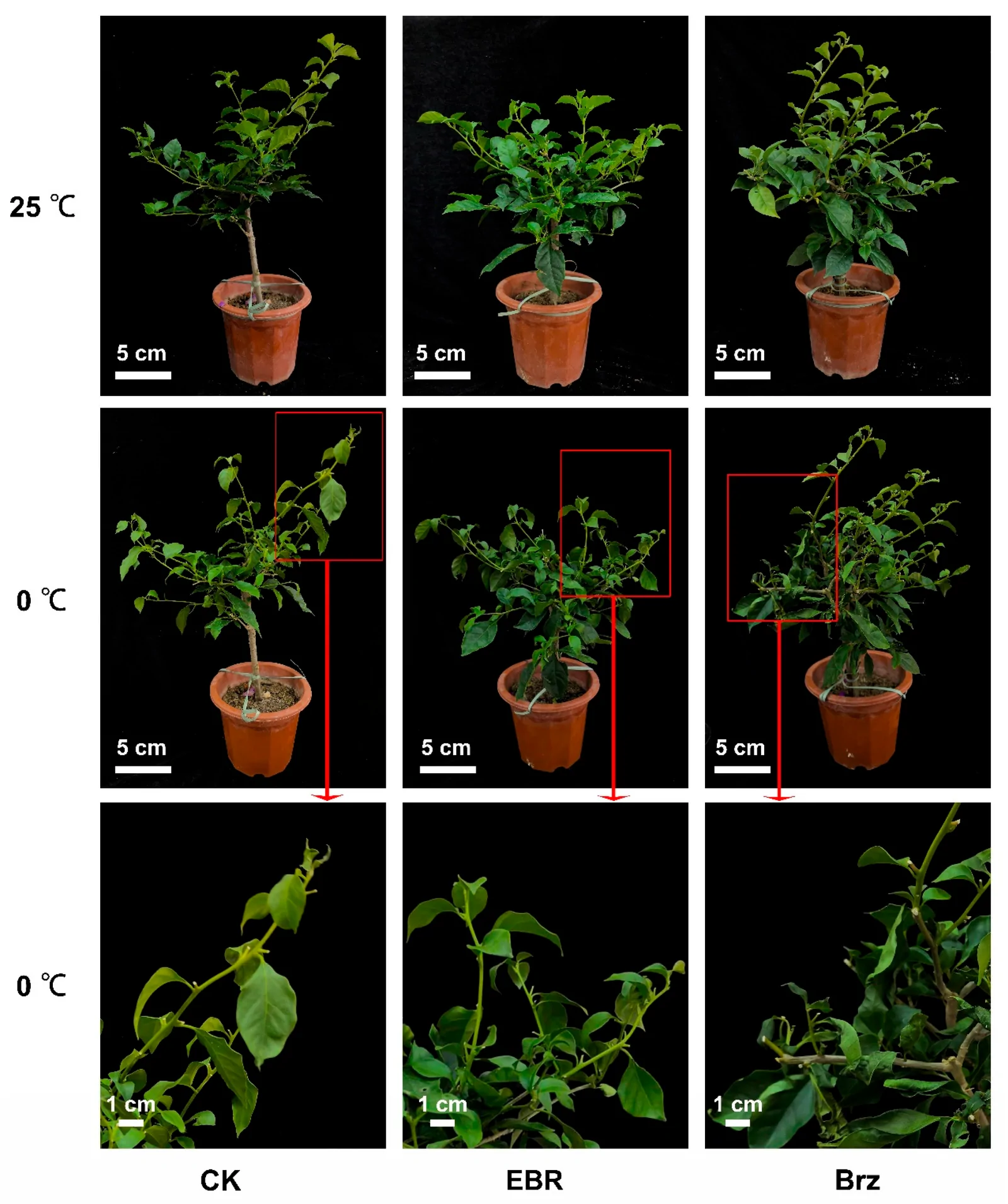
Exogenous EBR application improves cold tolerance in *Bougainvillea.* The potting plant picture, scale bar = 5 cm. The enlarged local image, scale bar = 1 cm.

To investigate the relationship between brassinosteroid (BR) signaling and the cold stress response in *Bougainvillea*, the *Bougainvillea* cultivar ‘Angus’ was treated with exogenous 24-epibrassinolide (EBR) and the BR biosynthesis inhibitor brassinazole (Brz). After 5 days of cold stress at 0 °C, plants in the CK group exhibited obvious leaf softening, wilting and drooping, showing severe cold injury symptoms. In contrast, plants subjected to EBR treatment displayed only slight leaf drooping and maintained a relatively healthy growth status, with markedly improved cold tolerance. By comparison, Brz-treated plants suffered the most severe damage, characterized by shrunken and dehydrated leaves, which represented typical freezing phenotypes (Figure 1).

**Figure 2:**
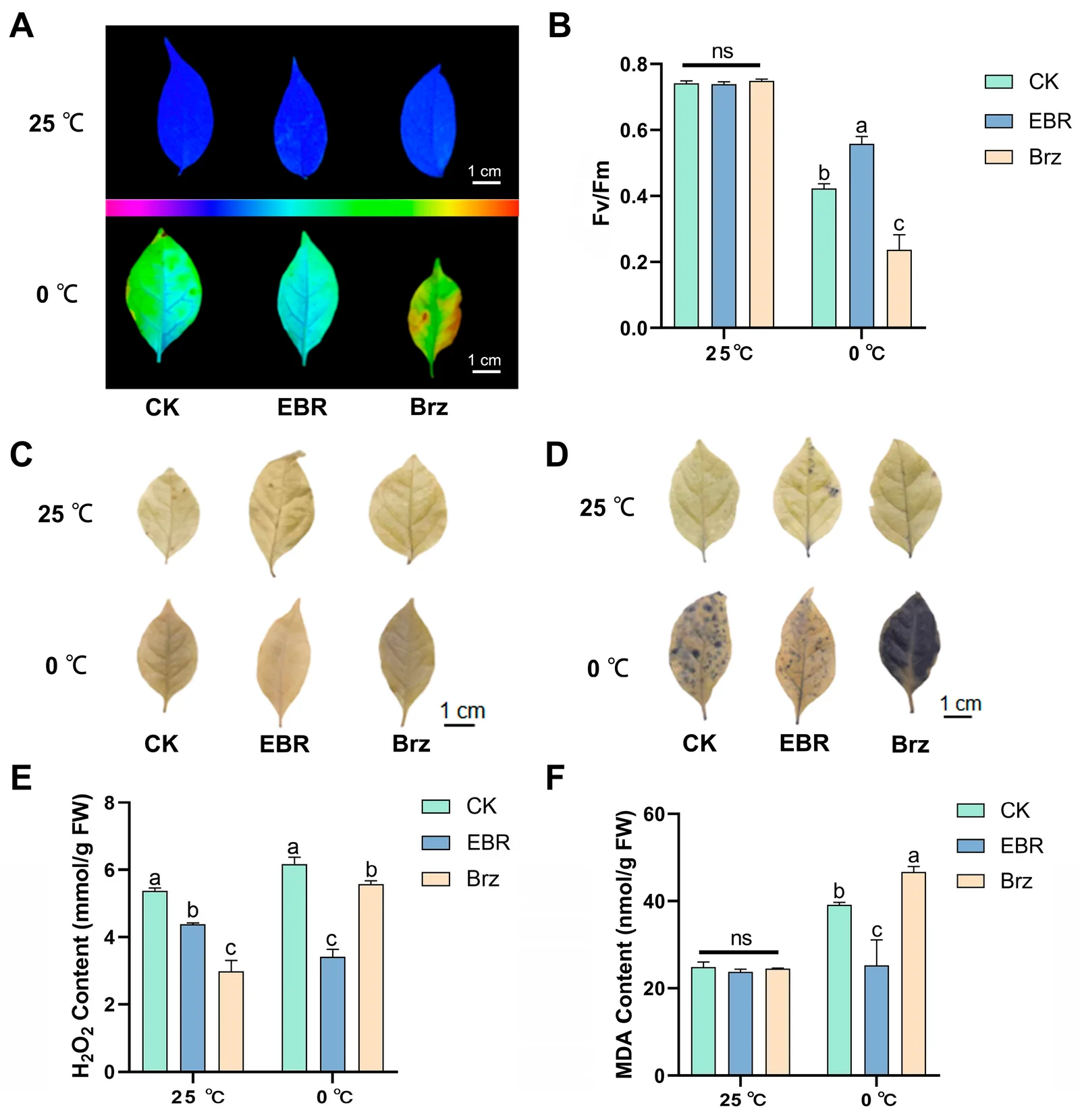
Exogenous EBR application significantly alleviates cold-induced leaf damage in *Bougainvillea.* (**A**) Chlorophyll fluorescence imaging. (**B**) Fv/Fm values. (**C**) DAB staining. (**D**) NBT staining. (**E**) H_2_O_2_ content. (**F**) MDA content. Data are mean ± SD (*n* = 3). ns, no significant difference. Different letters denote significant differences (*p* < 0.05, one-way ANOVA). Scale bar = 1 cm.

Chlorophyll fluorescence detection results indicated that exogenous EBR treatment effectively alleviated cold-induced damage in *Bougainvillea* under cold stress (Figure 2A,B). Histochemical staining with DAB and NBT further revealed that the accumulation of H_2_O_2_ and O_2_^•−^ was significantly reduced in the leaves of EBR-treated *Bougainvillea* compared with the CK group, whereas opposite phenotypic changes were observed in Brz-treated plants (Figure 2C–E). Consistent with the above results, the determination of MDA content further verified that EBR application markedly mitigated cold stress-caused injury in *Bougainvillea* (Figure 2F).

**Figure 3:**
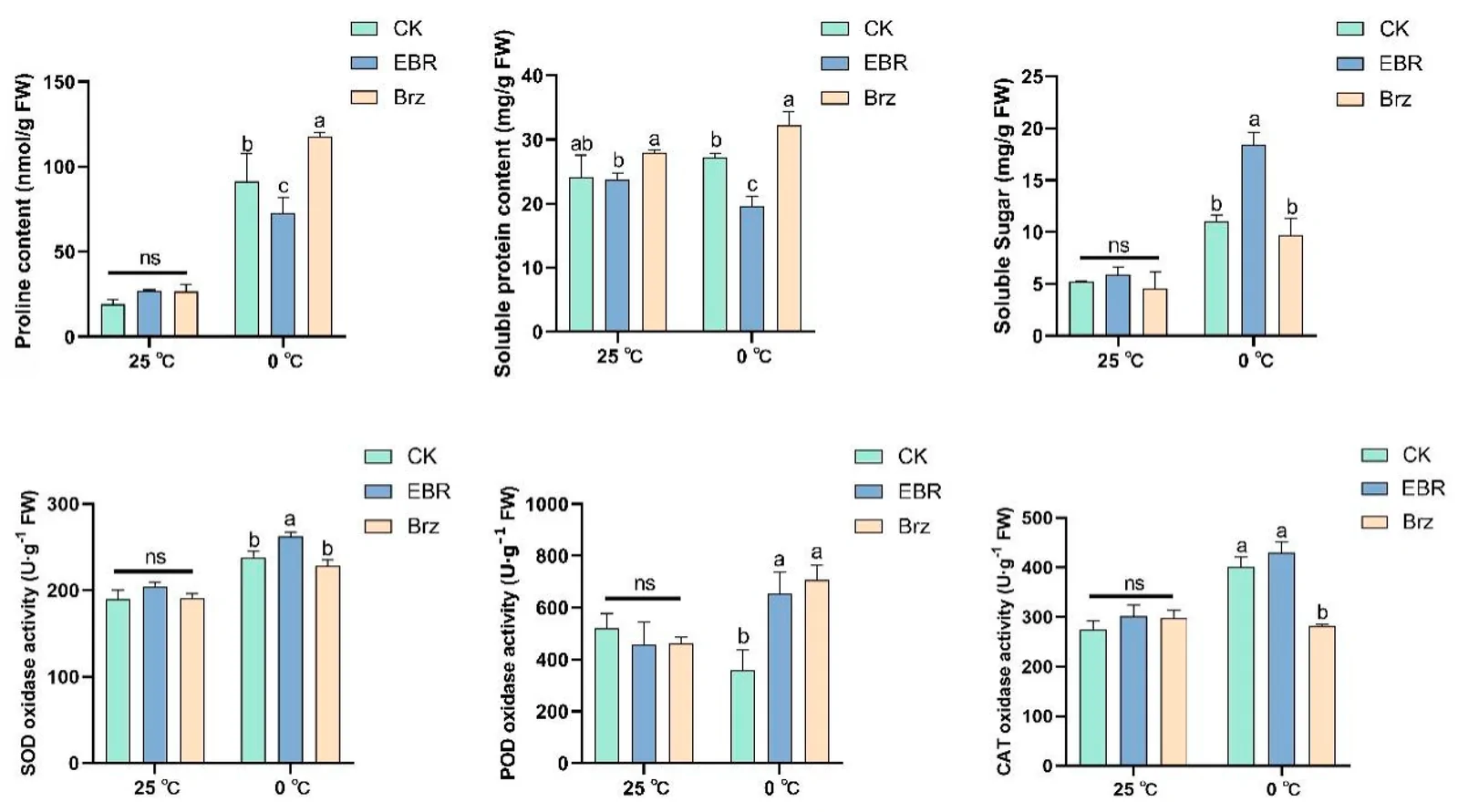
Effects of EBR and Brz on osmotic adjustment substances and antioxidant enzyme activities in *Bougainvillea.* Data are mean ± SD (*n* = 3). ns, no significant difference. Different letters denote significant differences (*p* < 0.05, one-way ANOVA).

To explore the effects of exogenous EBR application on physiological responses of *Bougainvillea* under cold stress, we determined the contents of osmotic adjustment substances (including proline, soluble protein, and soluble sugar) and the activities of antioxidant enzymes (SOD, POD, and CAT) (Figure 3). Under normal temperature conditions (25 °C), no significant differences in the contents of proline, soluble protein, and soluble sugar or in the activities of SOD, POD, and CAT were observed among the CK, EBR, and Brz treatment groups. However, cold stress markedly altered these physiological indicators with distinct treatment-specific differences.

Under low-temperature conditions, the proline content in the EBR-treated group was significantly lower than that in the CK group, while the Brz-treated group exhibited a remarkably higher proline accumulation relative to CK. For soluble protein, the EBR group showed a significantly lower soluble protein level than the CK group, whereas the Brz group possessed the highest soluble protein content among all treatments. In contrast, the soluble sugar content was significantly elevated in EBR-treated plants compared with CK, and the Brz group displayed a distinctly lower soluble sugar accumulation than the EBR group.

Antioxidant enzyme activity assays further revealed significant variations among treatments under cold stress. The EBR group exhibited the highest SOD activity, followed by the Brz group, while the CK group showed the lowest SOD activity. Both EBR and Brz treatments significantly increased POD activity compared with the CK group, with no significant difference detected between the two treatment groups. For CAT activity, the EBR group maintained the highest activity, followed by the CK group, whereas Brz treatment significantly inhibited CAT activity. Collectively, these results indicated that exogenous EBR application enhances the cold tolerance of *Bougainvillea* by increasing soluble sugar accumulation and promoting the activities of SOD and POD antioxidant enzymes under cold stress.

### 2.2. Identification of BpBZR1 Gene Family in Bougainvillea

A total of seven BpBZR1 family members were identified from the *Bougainvillea* genome using HMMER v 2.13.0 and BLASTp v 2.13.0 software. For subsequent systematic analysis, all identified BpBZR1 genes were named according to their sequence homology with *AtBZR1* genes in *Arabidopsis* (Table S1).

The physicochemical properties of BpBZR1s were analyzed using the ExPASy database (Table S1). The amino acid lengths of these proteins ranged from 285 residues (BpBEH4-2) to 676 residues (BpBZR3). The molecular weights of the seven BpBZR1 proteins varied between 30,561.12 Da (BpBEH4-2) and 76,228.18 Da (BpBZR3), with isoelectric points (pI) spanning from 5.22 (BpBZR2) to 9.2 (BpBEH2). Their instability indices ranged from 36.09 (BpBZR3) to 74.35 (BpBZR1), aliphatic indices fluctuated between 54.82 (BpBEH2) and 74.74 (BpBZR2), and grand average of hydropathicity (GRAVY) values fell in the scope of −0.652 (BpBEH2) to −0.435 (BpBEH3). In addition, the seven members of the BpBZR1 family were unevenly distributed on chromosomes 2, 3, 7, 14 and 16 of the *Bougainvillea* genome.

### 2.3. Phylogenetic Classification and Analysis of BpBZR1 Gene Family in Bougainvillea

**Figure 4:**
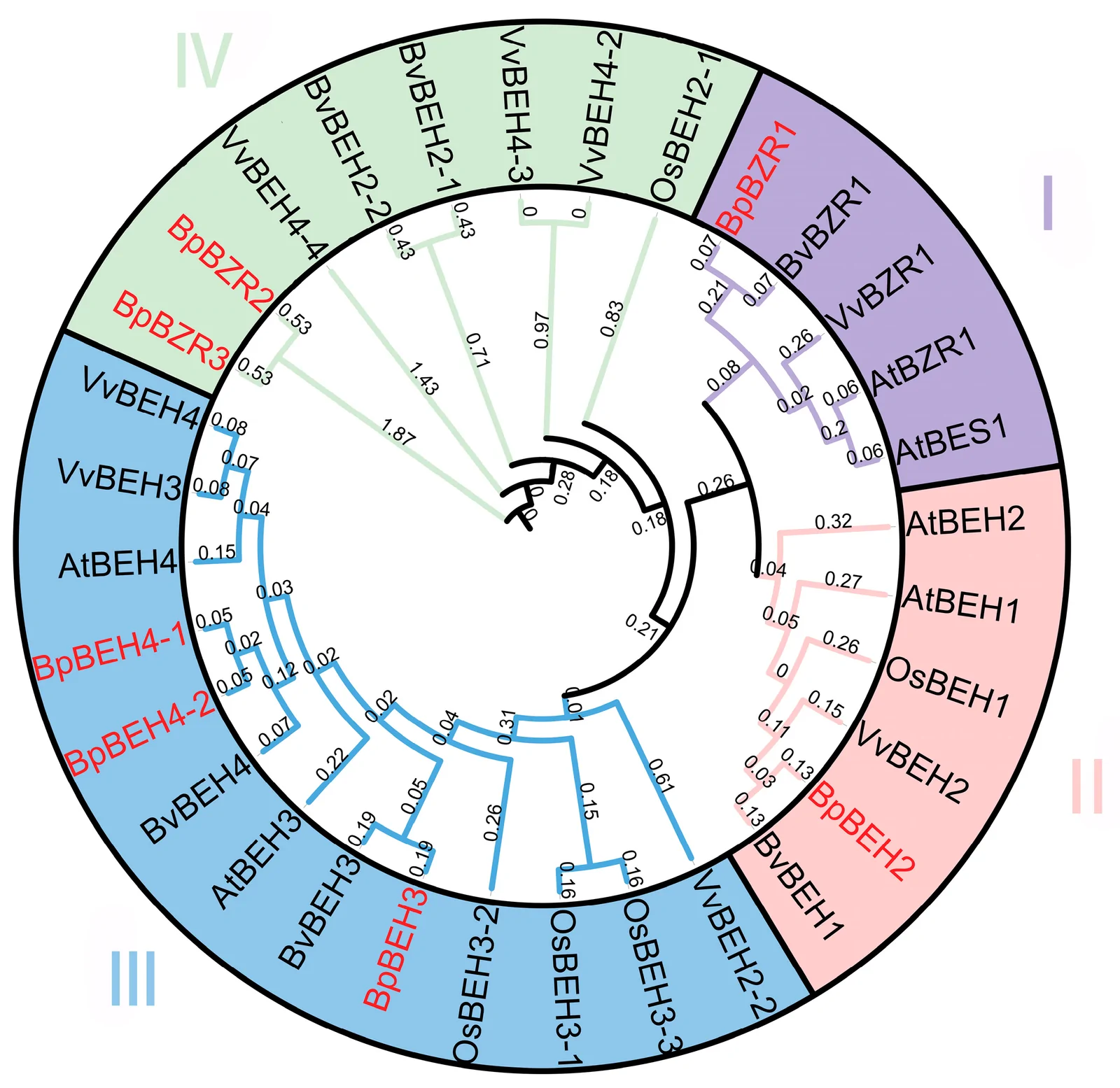
Phylogenetic tree analysis of members of the BZR1 gene family among five plant species.

To elucidate the evolutionary relationships among BpBZR1 genes, a phylogenetic tree was constructed using BZR-family protein sequences from *Bougainvillea*, *Arabidopsis thaliana*, *Vitis vinifera*, *Beta vulgaris* and *Oryza sativa* (Figure 4). Phylogenetic classification revealed that all identified BZR proteins were clustered into four distinct subgroups. Subgroup I harbored the largest number of members, including three BpBZR1 paralogs; one BpBZR1 was assigned to each of Subgroups II and III, whereas two BpBZR1s were distributed in Subgroup IV. Notably, no *Arabidopsis*-derived BZR1 members were detected within Subgroup IV.

### 2.4. Structural Analysis of the BpBZR1 Gene Family in Bougainvillea

**Figure 5:**
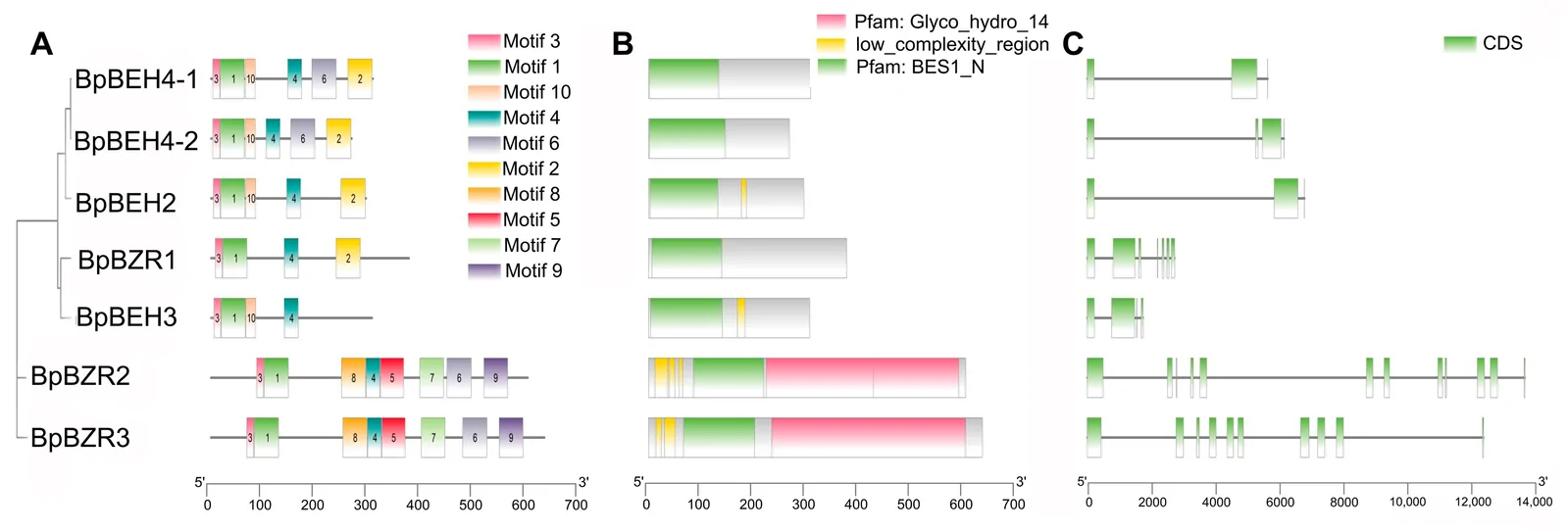
Phylogenetic relationships, conserved motifs, conserved domains and gene structures of BpBZR1 gene family members in *Bougainvillea*. (**A**) Phylogenetic tree and distribution of conserved motifs. (**B**) Prediction of conserved domains. (**C**) Gene structure of BpBZR1 family members.

Conserved motifs of the seven BpBZR1 proteins were identified via the MEME online tool, and a total of ten distinct motifs were predicted (Figure 5A). Conserved domain analysis verified that all characterized BpBZR1s possess the canonical BES_N domain, which further confirms the reliability of the identified BpBZR1 family members (Figure 5B). Based on the distribution patterns of conserved motifs and functional domains, these seven BpBZR1 proteins were categorized into two clades: members from the first clade contain only a single BES1_N domain, whereas proteins in the second clade carry both BES1_N and Glyco_HYHO_14 domains. Exon–intron structural profiling of the BpBZR1 genes further clustered the seven family members into three separate groups (Figure 5C). Specifically, BpBEH4-1, BpBEH4-2 and BpBEH2 were clustered into Group I; BpBZR1 together with BpBEH3 formed Group II; BpBZR2 and BpBZR3 constituted Group III. Genes within the same group exhibited highly conserved exon–intron organization (Figure 5C).

### 2.5. The Cis-Acting Elements of the BpBZR1 Genes Promoter in Bougainvillea

**Figure 6:**
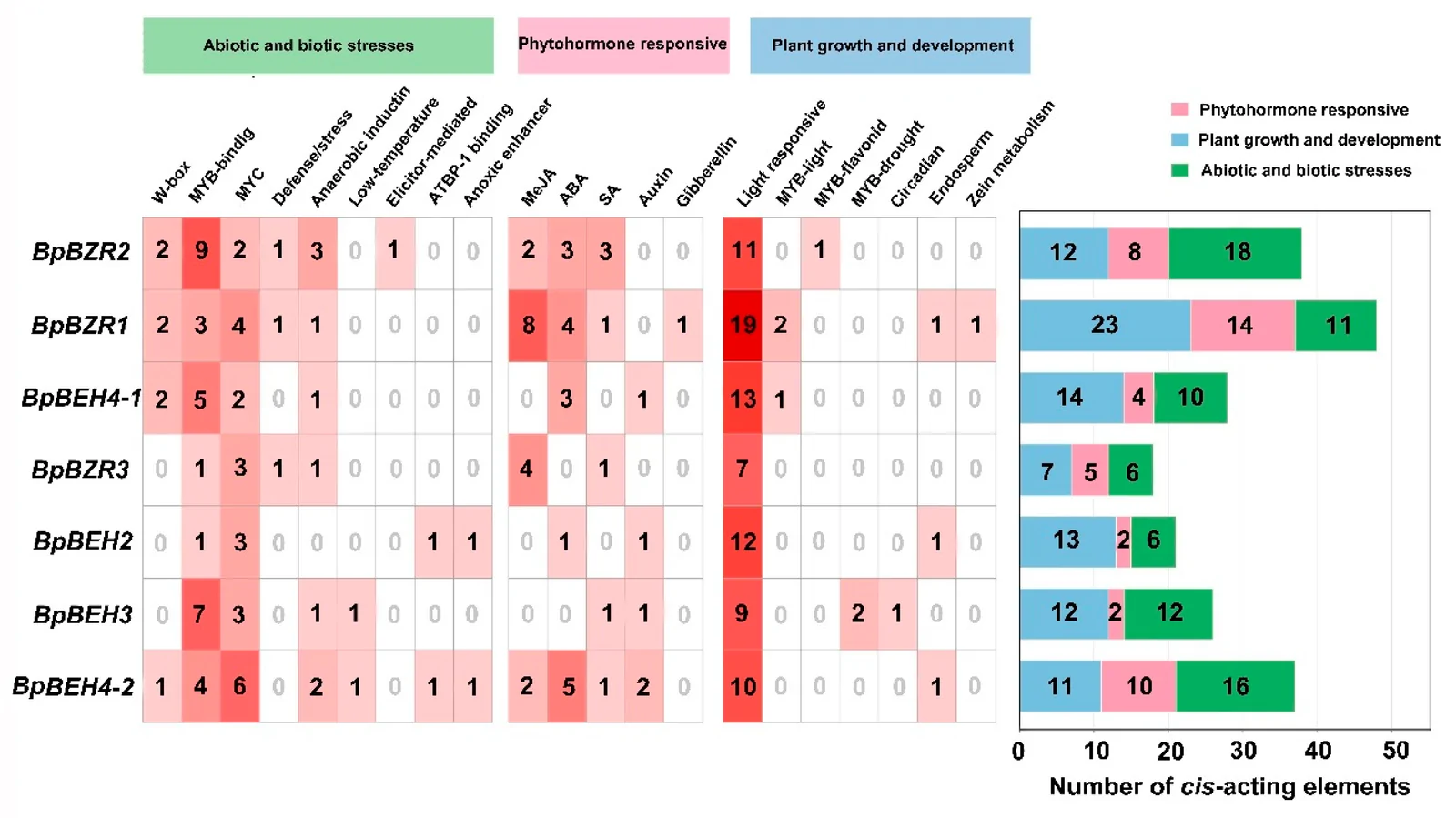
*Cis*-acting element analysis of BpBZR1 gene family promoters.

*Cis*-acting elements located in gene promoter regions serve as binding sites for transcription factors, and functional prediction of target genes can be achieved by characterizing these promoter *cis*-elements. In this study, *cis*-regulatory elements within the promoter sequences of *BpBZR1* genes were predicted using the PlantCARE online database, and numerous diverse *cis*-elements were identified. These predicted elements were functionally categorized into three major classes: those responding to biotic and abiotic stresses, phytohormone-responsive elements, and elements involved in plant growth and developmental processes (Figure 6). Substantial quantitative variations were observed among the three categories of *cis*-elements. Except for *BpBZR3*, the promoters of the remaining family members contained abundant growth- and development-related *cis*-elements, among which *BpBZR1* possessed the highest copy number (23 copies). This finding implies that *BpBZR1s* exert crucial regulatory functions during plant growth and development.

In addition, abundant stress-associated *cis*-elements, including W-box, low-temperature-responsive and ABA-responsive motifs, were widely distributed in these promoter regions. Five out of seven *BpBZR1* members (*BpBZR1*, *BpBZR2*, *BpBEH4-1, BpBEH3*, *BpBEH4-2*) harbored more than ten stress-related *cis*-elements in their promoter sequence. Notably, low-temperature-responsive elements were exclusively detected in the promoters of *BpBEH3* and *BpBEH4-2*, indicating the potential involvement of these two genes in cold stress responses.

### 2.6. Expression Pattern of BpBZR1s in Different Tissues and Under Cold Treatment

**Figure 7:**
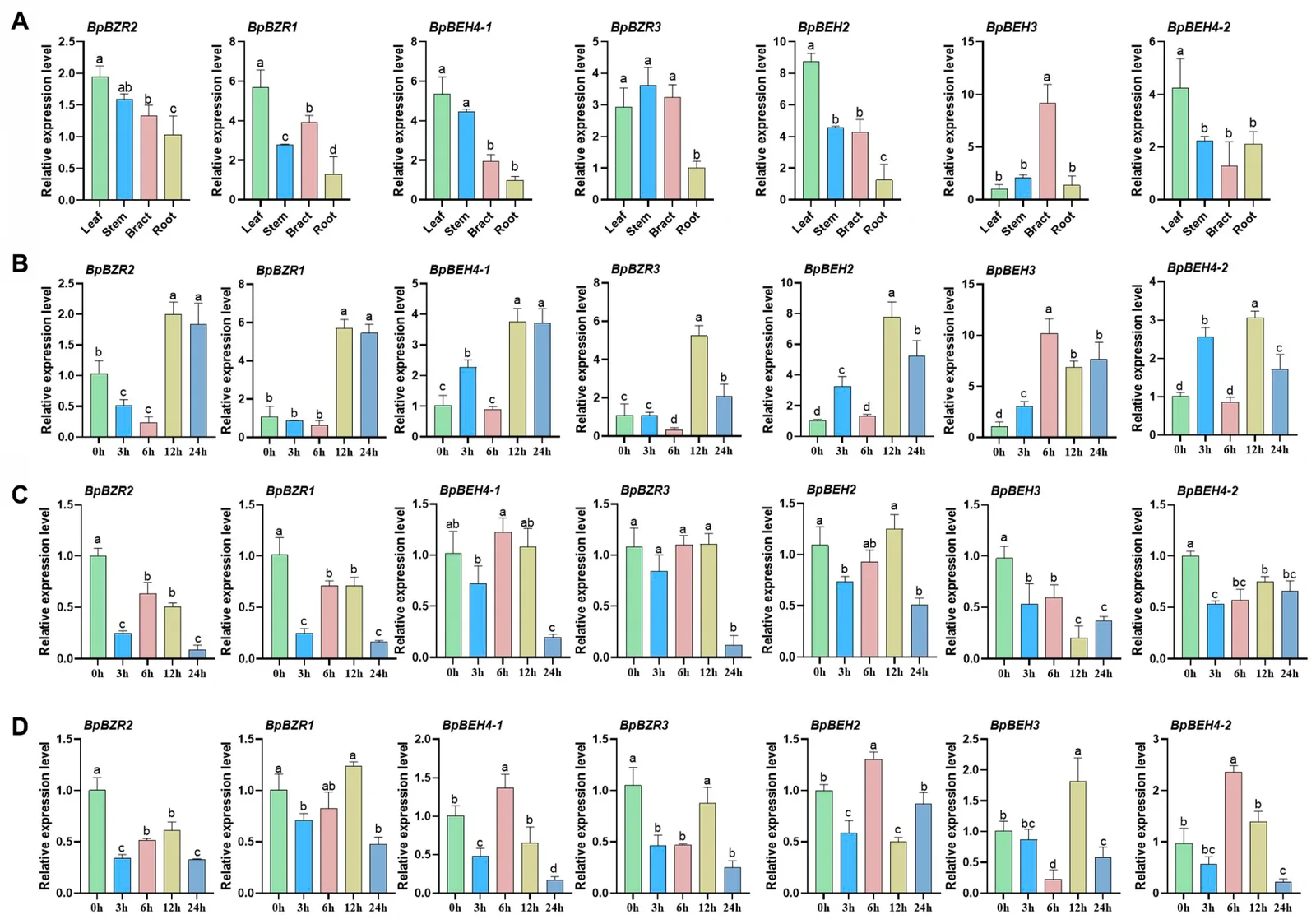
Expression pattern analysis of BpBZR1 gene family members. (**A**) Tissue-specific expression of BpBZR1 family genes in *Bougainvillea*. (**B**) Expression changes in BpBZR1 family genes after EBR treatment for 0, 3, 6, 12 and 24 h. (**C**) Expression changes in BpBZR1 family genes after exogenous Brz treatment for 0, 3, 6, 12 and 24 h. (**D**) Expression changes in BpBZR1 family genes under cold stress for 0, 3, 6, 12 and 24 h. Data are mean ± SD (*n* = 3). Different letters denote significant differences (*p* < 0.05, one-way ANOVA).

To dissect the expression characteristics of the BpBZR1 gene family in *Bougainvillea*, qRT-PCR was used to quantify the transcript abundance of seven family members across different tissues, under exogenous EBR, Brz and cold stress treatments. Tissue expression assays revealed that all seven genes were expressed in different tissues, and each has its own distinct tissue preference. *BpBZR2*, *BpBZR1*, *BpBEH4-1*, *BpBZR3*, *BpBEH2* and *BpBEH4-2* exhibited the highest expression in leaves, while *BpBEH3* was predominantly expressed in bracts, suggesting functional divergence among different members of the family (Figure 7A). Exogenous EBR significantly induced the transcription of most BpBZR1 family genes with a time-dependent upregulation pattern, and *BpBEH4-2* rapidly responded to BR signal at 6 h after EBR application (Figure 7B). In contrast, Brz, an inhibitor of BR biosynthesis, suppressed the expression of most family genes, and the transcript level of *BpBEH4-2* continuously decreased with prolonged Brz treatment (Figure 7C). Cold stress substantially reshaped the expression profiles of BpBZR1 family genes. *BpBEH4-2* was rapidly induced and peaked at 6 h of cold exposure before gradual decline, identifying it as an early cold-responsive gene (Figure 7D). These results indicate that *BpBEH4-2* participates in BR-mediated cold response in *Bougainvillea*.

### 2.7. BpBEH4-2 Is a Nuclear-Localized Member of the BES/BZR Transcription Factor Family

**Figure 8:**
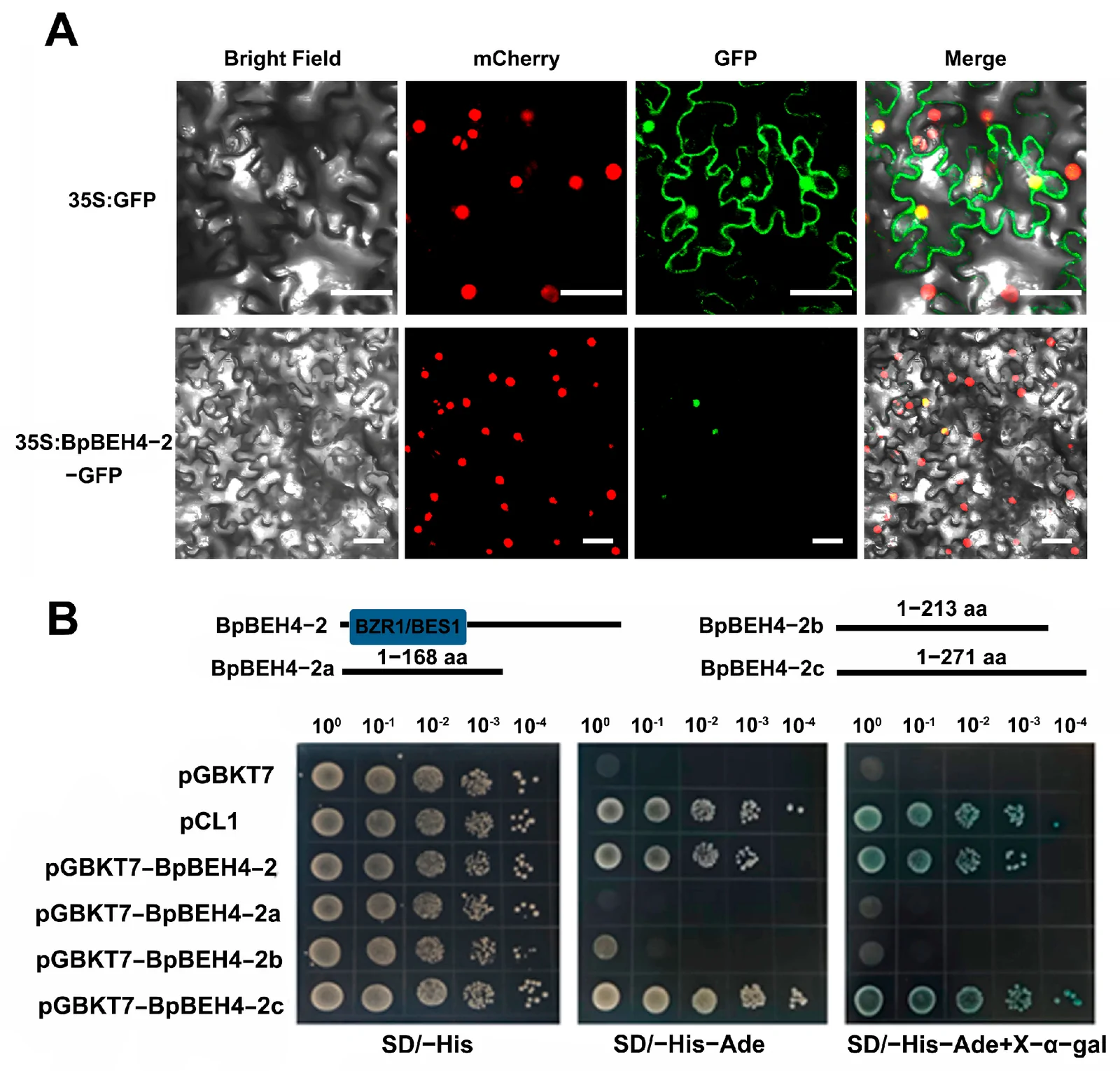
Multiple sequence alignment, subcellular localization and transcriptional autoactivation assay of BpBEH4-2 protein. (**A**) Subcellular localization of BpBEH4-2 in *Nicotiana benthamiana* epidermal cells. Scale bar = 50 μm. (**B**) Yeast autoactivation assay of BpBEH4-2.

Subcellular localization assay demonstrated that the green fluorescence signal of the 35S:BpBEH4-2-GFP fusion protein fully overlapped with the mCherry nuclear marker signal, indicating that BpBEH4-2 localizes to the nucleus, which is consistent with the subcellular feature of transcription factors (Figure 8A). As a BZR-family transcription factor, BpBEH4-2 may own intrinsic transcriptional activation capacity. The yeast self-activation assay is essential to verify its transcriptional regulatory property and map the functional activation domain. To map the transcriptional activation domain of BpBEH4-2, we designed three truncated fragments based on the position of the conserved BZR1/BES1 N-terminal DNA-binding domain: BpBEH4-2a (1-168 aa, containing only the N-terminal conserved BZR1/BES1 domain), BpBEH4-2b (1-213 aa, containing the conserved domain plus partial intermediate sequence), and BpBEH4-2c (1-271 aa, covering the complete C-terminal region). Yeast autoactivation assays showed that yeast strains transformed with full-length pGBKT7-BpBEH4-2 and truncated fragment BpBEH4-2c grew normally on SD/-His-Ade selective medium and turned blue on plates supplemented with X-α-gal, showing the same phenotype as the positive control pCL1. In contrast, the negative control pGBKT7 and truncated fragments BpBEH4-2a and BpBEH4-2b failed to survive on this medium. These results demonstrate that BpBEH4-2 possesses transcriptional autoactivation activity, and its transcriptional activation domain resides in the C-terminal region of the protein (Figure 8B).

### 2.8. Overexpression of BpBEH4-2 Improves Chilling Tolerance in Arabidopsis

**Figure 9:**
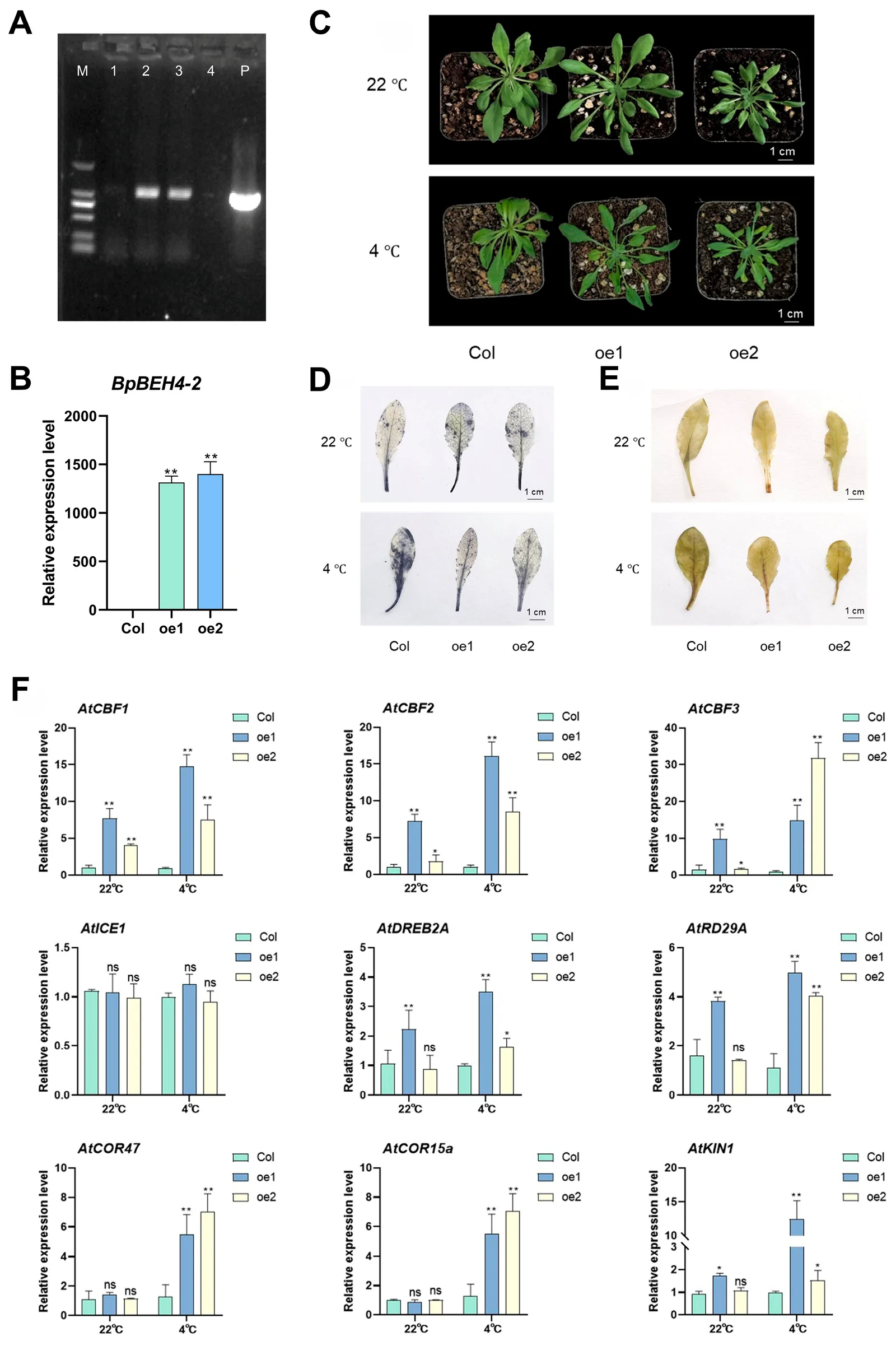
Overexpression of *BpBEH4-2* enhances chilling tolerance in transgenic *Arabidopsis*. (**A**) DNA-based identification of transgenic *Arabidopsis*-positive plants. (**B**) Identification of transgenic Arabidopsis plants that are positive by qRT-PCR. (**C**) Phenotypes of wild-type (*Col*) and *BpBEH4-2*-overexpressing transgenic *Arabidopsis* plants under cold stress. (**D**) NBT staining. (**E**) DAB staining. (**F**) qRT-PCR analysis of the expression levels of cold stress-related genes. Data are mean ± SD (*n* = 3). ns, no significant difference. Asterisk (*) indicates statistical significance at *p* < 0.05 and double asterisk (**) indicates statistical significance at *p* < 0.01 (*t*-test). Scale bar = 1 cm.

To characterize the potential regulatory function of *BpBEH4-2* in cold stress response, the open reading frame (ORF) of *BpBEH4-2* was cloned, and the overexpression construct pSAK277-*BpBEH4-2* was subsequently generated. Two independent *BpBEH4-2*-overexpressing (*BpBEH4-2*-oe) transgenic *Arabidopsis* lines were verified by PCR and qRT-PCR assays (Figure 9A,B). After low-temperature treatment, obvious leaf wilting, softening and collapse were observed in wild-type (*Col*) plants, whereas transgenic overexpression lines exhibited significantly enhanced chilling tolerance (Figure 9C). NBT and DAB histochemical staining were further performed to evaluate cold-induced oxidative damage. Under normal growth conditions before cold exposure, comparable faint staining was detected in the leaves of *Col* and transgenic plants, indicating similar basal reactive oxygen species (ROS) contents. After cold stress, extensive dark blue and brown precipitates were accumulated in *Col* leaves, which reflected a massive overproduction of O_2_^•−^ and H_2_O_2_. By contrast, significantly reduced NBT and DAB staining signals were observed in transgenic leaves, indicating that cold-induced oxidative damage was alleviated in the overexpression lines (Figure 9D,E). Collectively, these phenotypic assays confirmed that heterologous expression of *BpBEH4-2* substantially improves cold resistance in transgenic *Arabidopsis*.

qRT-PCR was applied to quantify the transcript levels of cold-responsive marker genes (Figure 9F). Before cold treatment, the transcriptional levels of *AtCBF1*, *AtCBF2* and *AtCBF3* in the BpBEH4-2-*oe* line were significantly higher than those in the *Col* line, and this expression difference further expanded after the cold treatment. No significant transcriptional differences in *AtDREB2A*, *AtRD29A*, *AtCOR47*, *AtCOR15a* and *AtKIN1* were detected between *Col* and transgenic plants under non-stressed conditions. However, their transcripts were drastically elevated in overexpression lines after cold treatment. In contrast, the expression of *AtICE1* remained unaffected by *BpBEH4-2* overexpression. These results further indicated that BpBEH4-2 functions as a positive regulator in the *Arabidopsis* cold stress signaling cascade.

## 3. Discussion

Cold stress is a major limiting factor restricting the large-scale cultivation and industrial planting of *Bougainvillea* in northern cold regions [5,18]. Exploring the molecular regulatory mechanisms of *Bougainvillea* in response to cold stress is critical for breeding cold-tolerant *Bougainvillea* cultivars. Brassinosteroids (BRs) are ubiquitous plant regulatory molecules that play essential roles in cold response, and exogenous BR application can markedly enhance plant cold tolerance [19]. It has been documented in multiple plant species, including *Arabidopsis* [10], banana [11] and grape [20]. Consistent with these previous findings, our phenotypic observations revealed that exogenous EBR treatment substantially alleviated cold-induced leaf wilting injury in *Bougainvillea*, whereas application of the BR biosynthesis inhibitor Brz exacerbated freezing damage. Under cold stress, EBR increased soluble sugar contents and activated the antioxidant enzymes SOD and POD, thereby strengthening cellular osmotic adjustment and ROS-scavenging capacity. Brz significantly decreased CAT activity, consistent with compromised cold tolerance. However, both Brz and EBR elevated SOD and POD activity relative to the control. Despite higher SOD and POD activities in Brz-treated plants, these plants still suffered severe cold injury, likely due to the loss of CAT-dependent ROS-scavenging capacity and disrupted osmotic adjustment.

As a key gene in the BR signaling pathway, *BZR1* has been confirmed to regulate plant growth, development and abiotic stress responses [21,22]. To date, genome-wide identification of the BZR1 gene family has been reported in multiple plant species, including *Brassica rapa* [23], *Brassica napus* [24], and *Malus pumila* Mill [25]. In this study, the published *Bougainvillea* genome data was used to conduct a comprehensive identification of the BZR1 gene family [26]. A total of seven *BpBZR1* genes were identified from the *Bougainvillea* genome. Phylogenetic tree analysis classified these BpBZR1 members into four subgroups. Notably, BpBZR2 and BpBZR3 clustered in Group IV contain two conserved domains, BES_N and Glyco_hydro_14. This structural characteristic has also been observed in tomato, cabbage, cotton and lotus [22,24,27]. In contrast, BZR1 family genes in maize, rice and *Arabidopsis* harbor only one conserved domain, BES_N [19,28]. Gene structure is closely associated with gene function, and phylogenetically related genes generally exhibit similar gene structures and conserved motifs [29,30]. Structural analysis of *BpBZR1s* revealed that genes within the same subgroup are highly conserved in the type and number of conserved motifs, gene length, and exon–intron organization, indicating that *BZR1* genes in the same subgroup may possess similar biological functions.

Accumulating studies have demonstrated that the BZR1 gene family positively regulates plant responses to cold stress [19]. For example, MdBES1 modulates the expression of *MdMYB88* to positively enhance cold stress tolerance in apple [31]. In melon, *CmBES1-1*, *CmBES1-3*, *CmBES1-5* and *CmBES1-6* exhibit significant expression changes under cold treatment [32]. Accordingly, this study focused on the functional characterization of BpBZR1s in mediating cold stress responses in *Bougainvillea*. Promoter *cis*-element analysis revealed that the promoters of *BpBZR2*, *BpBZR1*, *BpBEH4-1*, *BpBEH3* and *BpBEH4-2* contain numerous stress-responsive *cis*-elements. Notably, *BpBEH3* and *BpBEH4-2* possess specific low-temperature-responsive elements in their promoter regions. Furthermore, qRT-PCR validation confirmed that the expression of BpBEH4-2 is significantly induced by cold stress. Combined with the low-temperature-responsive cis-elements identified in its promoter, these findings imply that BpBEH4-2 is potentially involved in cold stress adaptation in *Bougainvillea*.

To further explore the potential regulatory function of BpBEH4-2 in cold stress response, we constructed the overexpression vector pSAK277-BpBEH4-2 and transformed it into *Arabidopsis* for a heterologous functional assay. Phenotypic results showed that transgenic *Arabidopsis* plants overexpressing *BpBEH4-2* exhibited enhanced chilling tolerance compared with wild-type (*Col*) plants. qRT-PCR analysis demonstrated that cold-responsive genes including *AtCBF1*, *AtCBF2*, *AtCBF3*, *AtDREB2A*, *AtRD29A*, *AtCOR47*, *AtCOR15a*, and *AtKIN1* were significantly increased in *BpBEH4-2* overexpression lines under low-temperature conditions. These heterologous data in *Arabidopsis* support that BpBEH4-2 can act as a positive regulator for plant chilling responses. This regulatory pattern is consistent with previous studies in *Arabidopsis*, apple, and tea plant [15,31,33].

Based on the available literature and our analysis, we demonstrated the positive function of BR in regulating cold stress responses in *Bougainvillea* and characterize the role of BpBEH4-2 in cold stress adaptation. Under cold stress, *BpBEH4-2* is activated in response to cold stress and BR signals, and enhances cold tolerance by upregulating the expression of cold-responsive genes, including *CBFs* and *CORs*. Nevertheless, several limitations remain in the present study. In future work, we aim to further validate these findings in *Bougainvillea* to corroborate the main conclusions obtained from *Arabidopsis* assays. Moreover, we will dissect the detailed regulatory network of BpBEH4-2 underlying cold stress responses in *Bougainvillea*.

## 4. Materials and Methods

### 4.1. Plant Materials and Treatment

The experimental materials used in this study were obtained from the Laboratory of Horticultural Plant Genetics and Breeding at Fujian Agriculture and Forestry University. The plant material consisted of the *Bougainvillea* var ‘Angus’. Wild-type *Arabidopsis thaliana* plants were grown in a growth chamber (seeds were preserved in our laboratory).

For 24-epibrassinolide (EBR) and brassinazole (Brz) treatments, the adaxial and abaxial surfaces of *Bougainvillea* leaves were uniformly sprayed with 1 mg/L EBR and 1 μM Brz solution, respectively. For low-temperature treatment, *Bougainvillea* cuttings were placed in a 0 °C environment, and leaves were collected at 6 h, 12 h, 24 h, 3 d, and 5 d after the onset of cold stress. The collected samples (with at least three different cuttings taken as biological replicates) were immediately frozen in liquid nitrogen (LN_2_) and stored at −80 °C.

### 4.2. Physiological and Biochemical Measurements

The IMAGING-PAM chlorophyll fluorescence imaging system was applied to measure the maximum photochemical efficiency of photosystem II (Fv/Fm) under dark-adapted conditions. Three biological replicates were set for all measurements, with three technical replicates included in each biological replicate. For histochemical staining, plant tissues were incubated in darkness with staining solutions containing 1 mg/mL nitro blue tetrazolium (NBT) or 3,3’-diaminobenzidine (DAB) [34].

Proline content was quantified via the ninhydrin colorimetric method. Briefly, 0.5 g of fresh tissue sample was weighed and extracted with sulfosalicylic acid in a boiling water bath. After cooling, the mixture was centrifuged to collect the supernatant. The supernatant was then reacted with ninhydrin solution, and absorbance was determined at 520 nm. Malondialdehyde (MDA) concentration was measured using the thiobarbituric acid (TBA) method. Approximately 1 g of sample was homogenized with trichloroacetic acid (TCA), followed by centrifugation to obtain the MDA test solution. The supernatant was incubated with TBA reagent in a boiling water bath, and absorbance was recorded at 532 nm. H_2_O_2_ content was detected in accordance with the manufacturer’s instructions of a Plant Hydrogen Peroxide (H_2_O_2_) Detection Kit (Nanjing Jiancheng Bioengineering Institute, Nanjing, Jiangsu, China). Soluble sugar (SS) content was detected in accordance with the manufacturer’s instructions of a soluble sugar assay kit (Nanjing Jiancheng Bioengineering Institute, Nanjing, Jiangsu, China). Soluble protein (SP) content was detected in accordance with the manufacturer’s instructions of a soluble protein assay kit (Nanjing Jiancheng Bioengineering Institute, Nanjing, Jiangsu, China).

Identical sample pretreatment procedures were adopted for the activity assays of CAT, POD and SOD [35]. Briefly, 0.1 g of fresh sample was extracted with phosphate-buffered saline (PBS), and the resulting supernatant was collected for subsequent determination of the three antioxidant enzyme activities. CAT activity was quantified by monitoring absorbance changes at 240 nm, as hydrogen peroxide exhibits strong light absorption at this wavelength. POD activity was measured via the guaiacol colorimetric method. POD catalyzes the oxidation of guaiacol into tetraguaiacol, which shows a maximum absorption peak at 470 nm. SOD activity was indirectly quantified by evaluating the reduction level of NBT under light illumination.

### 4.3. Identification of BpBZR1 Gene Family in Bougainvillea

The *BZR1* gene sequences in *Bougainvillea* were identified by querying the conserved BES1_N (PF05687) domain model using hmmer-4.13.0 software. The domain model was downloaded from the Pfam database (http://pfam.xfam.org (accessed on 20 August 2025)) [36]. The *Bougainvillea* genome data used in this study are available under BioProject accession number CNP0004115 from the China National GeneBank (https://www.cngb.org/ (accessed on 10 January 2025)) [26]. The six *AtBZR1* genes from the *Arabidopsis thaliana* genome were retrieved from The *Arabidopsis* Information Resource (https://www.arabidopsis.org/ (accessed on 20 August 2025)) with the following gene IDs: At1G19350, At1G75080, At3G50750, At4G36780, At4G18890, and At1G78700. Based on these *Arabidopsis* BZR1 sequences, all *BZR1* genes in the *Bougainvillea* genome were further identified using BLASTp-4.13.0. To validate the identified *BZR1* genes, the amino acid sequences of the extracted *Bougainvillea* BZR1 genes were analyzed for conserved domains using the Simple Modular Architecture Research Tool (SMART: http://smart.embl-heidelberg.de (accessed on 25 August 2025)) [37].

### 4.4. Phylogenetic Analysis and Classification of BZR Genes

The full-length amino acid sequences of BZR1 proteins from four species—*Arabidopsis thaliana*, *Beta vulgaris*, *Oryza sativa*, *Vitis vinifera*, and *Bougainvillea*—were aligned using MUSCLE software with default parameters. A neighbor-joining (NJ) phylogenetic tree was constructed with MEGA 7.0 software using 1000 bootstrap replications [38]. The BZR1 transcription factors were classified into distinct groups based on the topological structure of the phylogenetic tree.

### 4.5. The Gene Structures of BpBZR1s in Bougainvillea

The length and positional information of the *Bougainvillea* BZR1 genes were extracted from the GFF file of the *Bougainvillea* genome. The molecular weight (MW) and isoelectric point (pI) of the BZR1 proteins were calculated using the ProtParam tool on the ExPASy Server (https://web.expasy.org/protparam/ (accessed on 1 September 2025)). Protein motifs of BZR1 across subgroups were detected using the MEME online tool (https://meme-suite.org/meme/ (accessed on 1 September 2025)) to identify conserved motifs shared among BZR1 proteins. The resulting MAST XML file was downloaded and visualized using TBtools II v2.487 [39]. The gene structures of BpBZR1, including introns and exons, were analyzed with TBtools II.

### 4.6. Structural Analysis of Promoter Cis-Acting Elements

The obtained promoter sequences of *BpBZR1* genes were submitted to the PlantCARE database (http://bioinformatics.psb.ugent.be/webtools/plantcare/html/ (accessed on 8 September 2025)) for prediction of *cis*-acting regulatory elements [40].

### 4.7. RNA Isolation, cDNA Synthesis and Quantitative Real-Time PCR Analysis

Total RNA was extracted from *Bougainvillea* using the FastPure Universal Plant Total RNA Isolation Kit (Vazyme Biotechnology, Nanjing, China) according to the manufacturer’s instructions. The integrity of the total RNA was assessed by 1% agarose gel electrophoresis, and its concentration was measured using a NanoDrop 1000 spectrophotometer (Thermo, Waltham, Massachusetts, USA). First-strand cDNA was synthesized from 1 μg of total RNA per 20 μL reaction using the HiScript III RT SuperMix for qPCR (gDNA wiper) kit (Vazyme, Nanjing, China). The cDNA products were diluted 10-fold with deionized water prior to use. Quantitative real-time PCR (qRT-PCR) was performed on CFX96 Touch™ Real-Time PCR Detection System (Bio-Rad, Heracles, USA) using the ChamQ SYBR qPCR Master Mix kit (Vazyme, Nanjing, China). Each reaction contained 2 μL of diluted cDNA, with other components and conditions following the manufacturer’s protocol. The primer sequences used for qRT-PCR are listed in Supplementary Table S2. The *Bg18S* gene from *Bougainvillea* was used as an internal reference [41]. Each treatment included three biological replicates and three technical replicates. Relative expression levels were calculated using the 2^−ΔΔCt^ method [42].

### 4.8. Gene Cloning of BpBEH4-2 and Its Heterologous Expression in Arabidopsis

The *BpBEH4-2* gene sequence was amplified using the 2× Hieff Canace^®^ Plus PCR Master Mix (With Dye) (Yeasen Biotech Co. Shanghai, China). The PCR reaction was performed in a 50 μL volume under the following conditions: initial denaturation at 98 °C for 2 min; 40 cycles of denaturation at 98 °C for 10 s, annealing at 60 °C for 20 s, and extension at 72 °C for 30 s; followed by a final extension at 72 °C for 5 min. The PCR products were separated by 1% agarose gel electrophoresis, and the target fragment was excised and purified. After recovery, ligation, transformation, and sequencing, the CDS of *BpBEH4-2* was successfully obtained.

The coding sequence (CDS) of *BpBEH4-2* was assembled into the overexpression vector pSAK277 using a homologous recombination method. The verified recombinant plasmid was introduced into *Agrobacterium tumefaciens* GV3101 via the freeze–thaw method. The transformed bacteria were cultured in 50 mL of LB liquid medium supplemented with 100 mg/L kanamycin and 50 mg/L rifampicin at 28 °C with shaking at 200 rpm for 48 h. The bacterial cells were collected by centrifugation at 4000 rpm for 10 min and resuspended in an infiltration buffer (0.05% Silwet-77, 5% sucrose, and 1/2 MS liquid medium). After three rounds of infiltration, *Arabidopsis* seeds were collected and screened for positive BpBEH4-2 transgenic plants [43]. Positive transformants were identified using PCR and qRT-PCR. Seeds of the confirmed positive lines were germinated on 1/2 MS plates, and two-week-old seedlings were transplanted into soil. Primers used for cloning and vector construction are listed in Table S2.

### 4.9. Subcellular Localization

The experimental method was based on previous studies [44]. For subcellular localization of the protein in *Nicotiana benthamiana*, the CDS of *BpBEH4-2* without the stop codon was inserted into the pCambia1300-35S:GFP vector containing the CaMV 35S promoter and a GFP tag. Clone primers are listed in Table S2. Recombinant plasmids and empty vectors were transformed into *Agrobacterium tumefaciens* GV3101, followed by infiltration into the leaves of *Nicotiana benthamiana*. After 1 d dark incubation and 2 d light culture, RFP nuclear localization and GFP fluorescence signals were observed and imaged using scanning confocal microscope (Leica TCS SP8, Wetzlar, Germany). The empty vector was used as the negative control.

### 4.10. Transcriptional Activation Activity Analysis

The full-length CDS and truncated fragments of *BpBEH4-2* were separately inserted into the yeast expression vector pGBKT7, with empty pGBKT7 used as the negative control. The vector pCL1 containing the full-length *GAL4* coding sequence was adopted as the positive control. These constructs were transformed into yeast strain Y2H. Transformants harboring pCL1 were screened on SD/-Leu medium, while transformants of empty pGBKT7 and pGBKT7-BpBEH4-2 constructs were cultured on SD/-His medium at 30 °C for 3 days. Subsequently, the yeast transformants were transferred onto SD/-His-Ade medium supplemented with or without X-α-Gal for further screening and color identification.

### 4.11. Statistical Analysis

Data visualization and analysis were performed using GraphPad Prism version 7.00. Experimental data are presented as mean ± standard deviation (SD). Statistical significance was analyzed using SPSS 20.0 software (IBM Corp., Armonk, NY, USA). Differences between means were assessed using Student’s *t*-test or one-way analysis of variance (ANOVA). Individual differences were evaluated with Duncan’s test. A *p*-value ≤ 0.05 was considered statistically significant.

## Data Availability

Data will be made available on request.

## Supplementary Materials

The following supporting information can be downloaded at: https://www.mdpi.com/article/10.3390/plants15213278/s1, Table S1: The detailed characteristics of BZR1 genes identified in *Bougainvillea*; Table S2: List of primers used in this study.

## References

[1] Abarca-Vargas R., Petricevich V.L. (2018). Bougainvillea genus: A review on phytochemistry, pharmacology, and toxicology. Evid.-Based Complement. Altern. Med..

[2] Fawad S.A., Khalid N., Asghar W., Suleria H.A.R. (2012). In vitro comparative study of *Bougainvillea spectabilis* “stand” leaves and *Bougainvillea variegata* leaves in terms of phytochemicals and antimicrobial activity. Chin. J. Nat. Med..

[3] Salam P., Bhargav V., Gupta Y., Nimbolkar P. (2017). Evolution in bougainvillea (*Bougainvillea Commers*.)—A review. J. Appl. Nat. Sci..

[4] Datta S. (2024). Literature survey and futuristic research approach on floriculture: *Bougainvillea*. Discov. Plants.

[5] Feng X., Hong Y., Fang Y., Lyu Y., Li H. (2025). Key Genes Related to the Cold Resistance of *Bougainvillea glabra* “Elizabeth Angus”. Russ. J. Plant Physiol..

[6] Saifuddin M., Khandaker M.M., Abdullah M.A., Mahmud K., Sajili M.H. (2017). Physiological stresses and phyto-hormones: Effects on the flowering process and longevity of *Bougainvillea*. Biosci. Res..

[7] Zhang Y., Yanfen N., Shengjing P. (2018). Role and Mechanism of Action of Exogenous Salicylic Acid and Sodium Molybdate in Improving Cold Tolerance of *Bougainvillea glabra*. Agric. Biotechnol. (2164-4993).

[8] Li Z., He Y. (2020). Roles of brassinosteroids in plant reproduction. Int. J. Mol. Sci..

[9] Anjum S., Wang L., Farooq M., Hussain M., Xue L., Zou C. (2011). Brassinolide application improves the drought tolerance in maize through modulation of enzymatic antioxidants and leaf gas exchange. J. Agron. Crop Sci..

[10] Kagale S., Divi U.K., Krochko J.E., Keller W.A., Krishna P. (2007). Brassinosteroid confers tolerance in *Arabidopsis thaliana* and Brassica napus to a range of abiotic stresses. Planta.

[11] Li D., Wu X., Li L., Wang Y., Xu Y., Luo Z. (2022). Epibrassinolide enhanced chilling tolerance of postharvest banana fruit by regulating energy status and pyridine nucleotide homeostasis. Food Chem..

[12] Chen G., Hou Y., Zheng Y., Jin P. (2022). 2, 4-epibrassinolide enhance chilling tolerance of loquat fruit by regulating cell wall and membrane fatty acid metabolism. Sci. Hortic..

[13] Eremina M., Unterholzner S.J., Rathnayake A.I., Castellanos M., Khan M., Kugler K.G., May S.T., Mayer K.F., Rozhon W., Poppenberger B. (2016). Brassinosteroids participate in the control of basal and acquired freezing tolerance of plants. Proc. Natl. Acad. Sci. USA.

[14] Li J.-W., Zhou P., Yang N., Hu Z.-H., Chen Y., Luo W., Kong J.-Y., Qin Z.-Y., Li X.-H., Chen X. (2024). CsBZR1 family transcription factors in wild and cultural tea plants and their response to hormone and abiotic stress. J. Plant Growth Regul..

[15] Li H., Ye K., Shi Y., Cheng J., Zhang X., Yang S. (2017). BZR1 positively regulates freezing tolerance via CBF-dependent and CBF-independent pathways in *Arabidopsis*. Mol. Plant.

[16] Zhao X., Ma K., Li Z., Li W., Zhang X., Liu S., Meng R., Lu B., Li X., Ren J. (2023). Transcriptome analysis reveals brassinolide signaling pathway control of foxtail millet seedling starch and sucrose metabolism under freezing stress, with implications for growth and development. Int. J. Mol. Sci..

[17] An S., Liu Y., Sang K., Wang T., Yu J., Zhou Y., Xia X. (2023). Brassinosteroid signaling positively regulates abscisic acid biosynthesis in response to chilling stress in tomato. J. Integr. Plant Biol..

[18] Zhang H., El-Kassaby Y.A., Zhou Q., Huang T., Sheng Q., Zhu Z. (2025). Integrative physiology, transcriptomic, and metabolomic analysis reveals key genes and pathways underlying chilling tolerance in *Bougainvillea*. Plant Physiol. Biochem..

[19] Li L., Mu T., Zhang R., Zhang G., Lyu J., Liu Z., Luo S., Yu J. (2025). The BES1/BZR1 family transcription factor as critical regulator of plant stress resilience. Plant Stress.

[20] Wang Y., Jiang Q., Wang X., Xi Z. (2023). Brassinosteroid Stimulates Hydrogen Peroxide Biosynthesis and Reduces the Effect of Cold Stress. J. Plant Growth Regul..

[21] Yang J., Wu Y., Li L., Li C. (2022). Comprehensive analysis of the BES1 gene family and its expression under abiotic stress and hormone treatment in *Populus trichocarpa*. Plant Physiol. Biochem..

[22] Zhou P., Jiang H., Li J., Jin Q., Wang Y., Xu Y. (2023). Genome-wide identification reveals that BZR1 family transcription factors involved in hormones and abiotic stresses response of lotus (*Nelumbo*). Horticulturae.

[23] Saha G., Park J.-I., Jung H.-J., Ahmed N.U., Kayum M.A., Kang J.-G., Nou I.-S. (2015). Molecular characterization of BZR transcription factor family and abiotic stress induced expression profiling in *Brassica rapa*. Plant Physiol. Biochem..

[24] Song X., Ma X., Li C., Hu J., Yang Q., Wang T., Wang L., Wang J., Guo D., Ge W. (2018). Comprehensive analyses of the BES1 gene family in Brassica napus and examination of their evolutionary pattern in representative species. BMC Genom..

[25] Jiang S., Li S., Liu X., Wen B., Wang N., Zhang R., Li D., Chen X., Fu X., Xiao W. (2021). Genome-wide identification and characterization of the MdBZR1 gene family in apple and their roles in improvement of drought tolerance. Sci. Hortic..

[26] Lan L., Zhao H., Xu S., Kan S., Zhang X., Liu W., Liao X., Tembrock L.R., Ren Y., Reeve W. (2023). A high-quality *Bougainvillea* genome provides new insights into evolutionary history and pigment biosynthetic pathways in the Caryophyllales. Hortic. Res..

[27] Su D., Xiang W., Wen L., Lu W., Shi Y., Liu Y., Li Z. (2021). Genome-wide identification, characterization and expression analysis of BES1 gene family in tomato. BMC Plant Biol..

[28] Sun Z., Liu X., Zhu W., Lin H., Chen X., Li Y., Ye W., Yin Z. (2022). Molecular traits and functional exploration of BES1 gene family in plants. Int. J. Mol. Sci..

[29] Liu H., Lyu H.-M., Zhu K., Van de Peer Y., Cheng Z.-M. (2021). The emergence and evolution of intron-poor and intronless genes in intron-rich plant gene families. Plant J..

[30] Lei P., Wei X., Gao R., Huo F., Nie X., Tong W., Song W. (2021). Genome-wide identification of PYL gene family in wheat: Evolution, expression and 3D structure analysis. Genomics.

[31] Liu X., Zhao C., Gao Y., Xu Y., Wang S., Li C., Xie Y., Chen P., Yang P., Yuan L. (2021). A multifaceted module of BRI1 ETHYLMETHANE SULFONATE SUPRESSOR1 (BES1)-MYB88 in growth and stress tolerance of apple. Plant Physiol..

[32] Zhang J., Liu L., Li W., Zhang J., Hao J. (2025). Genome-wide identification and expression analysis of BES1/BZR1 gene family in melon (*Cucumis melo* L.) and functional characteristics of CmBES1-3. Plant Physiol. Biochem..

[33] Wang C., Yang J., Zhao Y., Zhao D.-G. (2026). The CsBES1–14-CsCOR413 module mediated by brassinolide positively regulates cold resistance in tea plant. Hortic. Res..

[34] Sun X., Wang P., Jia X., Huo L., Che R., Ma F. (2018). Improvement of drought tolerance by overexpressing *MdATG18a* is mediated by modified antioxidant system and activated autophagy in transgenic apple. Plant Biotechnol. J..

[35] Wang S.-D., Zhu F., Yuan S., Yang H., Xu F., Shang J., Xu M.-Y., Jia S.-D., Zhang Z.-W., Wang J.-H. (2011). The roles of ascorbic acid and glutathione in symptom alleviation to SA-deficient plants infected with RNA viruses. Planta.

[36] Mistry J., Chuguransky S., Williams L., Qureshi M., Salazar G.A., Sonnhammer E.L., Tosatto S.C., Paladin L., Raj S., Richardson L.J. (2021). Pfam: The protein families database in 2021. Nucleic Acids Res..

[37] Letunic I., Khedkar S., Bork P. (2020). SMART: Recent updates, new developments and status in 2020. Nucleic Acids Res..

[38] Kumar S., Stecher G., Tamura K. (2016). MEGA7: Molecular evolutionary genetics analysis version 7.0 for bigger datasets. Mol. Biol. Evol..

[39] Chen C., Wu Y., Li J., Wang X., Zeng Z., Xu J., Liu Y., Feng J., Chen H., He Y. (2023). TBtools-II: A “one for all, all for one” bioinformatics platform for biological big-data mining. Mol. Plant.

[40] Lescot M., Déhais P., Thijs G., Marchal K., Moreau Y., Van de Peer Y., Rouzé P., Rombauts S. (2002). PlantCARE, a database of plant cis-acting regulatory elements and a portal to tools for in silico analysis of promoter sequences. Nucleic Acids Res..

[41] Kang Y., Li Y., Zhang T., Wang P., Liu W., Zhang Z., Yu W., Wang J., Wang J., Zhou Y. (2023). Integrated metabolome, full-length sequencing, and transcriptome analyses unveil the molecular mechanisms of color formation of the canary yellow and red bracts of *Bougainvillea × buttiana* ‘Chitra’. Plant J..

[42] Schmittgen T.D., Livak K.J. (2008). Analyzing real-time PCR data by the comparative CT method. Nat. Protoc..

[43] Clough S.J., Bent A.F. (1998). Floral dip: A simplified method for *Agrobacterium*-mediated transformation of *Arabidopsis thaliana*. Plant J..

[44] Jiang H., Zhou P., Jin Q., Wang Y., Liu F., Xu Y. (2024). Transcriptomics and metabolomics analysis revealed the regulatory network of apical buds development in lotus (*Nelumbo nucifera* Gaertn.). Sci. Hortic..

